# Incidence and correlates of low birth weight at a referral hospital in Northwest Ethiopia

**Published:** 2012-05-04

**Authors:** Berihun Megabiaw Zeleke, Meseret Zelalem, Nuru Mohammed

**Affiliations:** 1 Department of Epidemiology and Biostatistics, College of Medicine and Health Sciences, University of Gondar, Ethiopia; 2 Department of Paediatrics and Child Health, College of Medicine and Health Sciences, University of Gondar, Ethiopia; 3 Department of Gynaecology and Obstetrics, College of Medicine and Health Sciences, University of Gondar, Ethiopia

**Keywords:** Prevalence, Low Birth Weight, referral hospital, Ethiopia

## Abstract

**Background:**

Weight at birth is a good indicator of the newborn's chances for survival, growth, long-term health and psychosocial development. Low birth weight (LBW) babies are significantly at risk of death, contributing to the high perinatal morbidity and mortality in developing countries. Hence, this study aims to assess the incidence and associated factors of low birth weight (LBW) in Gondar University Hospital deliveries.

**Methods:**

A cross-sectional study, conducted on 305 live births from May 1- July 30, 2010. Information on independent variables was collected from the mothers just before discharge using a structured interview questionnaire. Neonatal weight was measured using standard beam balance. Both interviews and weight measurements were done by two trained midwives. Gestational age was determined by last normal menstrual period and/or ultrasound examinations.

**Results:**

The mean and standard deviations of the birth weights were 2976 ±476 grams. Incidence of LBW (birth weight <2500 grams) was 17.1% (95%CI 13.3%, 21.6%). LBW was associated with first delivery (AOR=2.85), lack of antenatal care follow up (AOR= 5.68) or infrequent visits and being HIV positive (AOR=3.22). More female newborns were with low birth weight than males though the difference was not significant after controlling for potential confounders in the multivariate analysis.

**Conclusion:**

There is a high incidence of LBW. Efforts should to enhance national antenatal care utilization in general, and particularly in Gondar, should be encouraged as its absence is closely associated with LBW.

## Background

The World Health Organization (WHO) defines Low Birth Weight (LBW) as having a weight of less than 2500 grams at birth [1]. LBW and preterm birth are major determinants of perinatal survival, infant morbidity and mortality as well as risk of developmental disabilities and illnesses throughout future lives [2, 3]. Birth weight has emerged as the leading indicator of infant health and welfare and the central focus of infant health policy [4]. This is because low birth weight (LBW) infants experience severe health problems and developmental difficulties that can impose enormous costs on families in particular and societies in general [5].

Babies born with a LBW are more likely to have health problems and slower development from immediately after birth to later in life [2, 6]. Lifelong problems include adult-onset diabetes, coronary heart disease, high blood pressure, intellectual, physical and sensory disabilities, and psychological and emotional distress [7]. LBW babies usually need extra hospital care, and there is a constant concern and uncertainty over future health outcomes. However, little attention is paid to birthweight improvement as a means of reducing child mortality [8].

An estimated 18 million babies worldwide are born each year with low birth weight of which about 3.1 million are in sub-Saharan Africa [1]. One of the goals of the 1990 World Summit for Children was to reduce the incidence of LBW to less than 10% by the year 2000. Despite this, LBW continues to remain a major public health problem in many sub-Saharan African countries where the incidence of LBW was estimated around 13% to 15% [2, 9]. About 70% of all LBW babies are born preterm, the remaining 30% at full term [10]. LBW is associated with many socio-economic factors such as residence (urban-rural difference), education, mother's age and occupation, birth order, the family's income and many maternal conditions such as nutritional status, tobacco use, mother's educational and health status [11–13].

Ethiopia, having an infant mortality rate of 59/1000 live births[14] has limited data on birth weight estimates as most deliveries take place at home leading to a highly biased maternal subjective inclusion of a “very small baby” in the reports [15]. Previous studies in Ethiopia reported a decline in mean birth weight and an increasing trend of LBW from 1970's to 1990's and 22.5% incidence of LBW among health institution deliveries in the south western part of the country [16]. Hence, this study aims to objectively assess the incidence and associated factors of LBW among neonates delivered at Gondar University referral hospital.

## Methods

### Study design and area

The institution based cross-sectional study was conducted at the University of Gondar Referral Hospital from May 1, 2010 through July 30, 2010. The study was conducted at the Maternity ward, Department of Gynecology and Obstetrics, University of Gondar Referral Hospital in Gondar town, Northwest Ethiopia.

The hospital is located in North Gondar Zone at about 727 kilometers Northwest of Addis Ababa (the capital city of Ethiopia). The hospital provides delivery services 24 hours a day, 7 days a week with its staff including midwives, intern doctors, eight general practitioners and three obstetricians.

### Sampling

A sample size of 309 was calculated using single proportion formula assuming a 22.5% proportion of LBW (from previous study in Ethiopia [16] at a 95% confidence limit, 80% power, 5% margin of error and adding 15% as contingency for non-response. A consecutive sampling technique (including all eligible participants) was employed till the calculated sample was achieved in the three month data collection period. All live births after 28 weeks of gestation at the hospital during the study period were included in the study. Multiple deliveries and deliveries of unknown gestational ages (unknown LNMP and no ultrasound estimation) were not included in this study.

### Data Collection

Data were collected by two regular time worker midwives in the delivery room. Training on the standard procedures of weight measurement and interview was provided to data collectors. Each newborn recruited was weighed only once, soon after delivery (within 2 hours). Maternal information was collected just before discharge. Birth weights and their codes were recorded together so as to provide a link to the maternal interview results.

### Study Variables

The interview questionnaire consisted of three parts (maternal socio-demography, Obstetric and Gynaecologic histories and variables related to the newborn). Mothers were asked about their obstetric and gynecologic histories, morbidity experiences and other relevant information using a structured interview questionnaire.

Relevant data such as history of medical or obstetric illnesses and laboratory profiles were retrieved from the antenatal (ANC) follow up charts when available, otherwise self reports by the mother were taken. Each mother was asked about her last normal menstrual period (LNMP) to calculate gestational age. Efforts were made to help mothers remember their LNMP dates by associating them with traditional and local events. For mothers who failed to remember or having unknown LNMP, gestational age estimated by ultrasound during pregnancy was taken.

Previous history of delivery of LBW babies was only subjectively assessed from the mothers speaking of “small or very small baby”. Dietary history was grossly assessed if the woman said she had additional dietary intake during the pregnancy and whether she was given counseling about the importance and types of additional food intake during pregnancy. Pretest was done on 15 deliveries just a day before the actual data collection. Weighing scales were checked and adjusted at zero level between each measurement.

### Data analysis

Data were entered and analyzed by SPSS version 16.0 statistical package for windows (SPSS Inc, Chicago). Logistic regression analyses were conducted to determine the effect of factor(s) on the outcome variable and to control possible confounders. Factors with a p-value < 0.05 were taken as statistically significant.

Ethical approval was obtained from the Institutional Review Board of the University of Gondar. Permission to conduct this study was also obtained from the medical director's office of the hospital. The purpose of the study was explained to the mothers and consent was obtained from them regarding their agreement to participate in the study. Names were not recorded in order to keep the identity of respondents anonymous.

## Results

A total of 305 neonate/mother pairs were involved in this study with a response rate of 98.7%. About 93% of the mothers were aged 18-35 years, the range between 15-41 years with a mean and SD of 25.37±5.29 years. More than three quarters were urban dwellers. Majorities were Amhara (97.0%) ethnics, married (93.8%) and Orthodox Christians (85.2%) (Table 1).

**Table 1:** Socio-demographic characteristics of study participants at Gondar University Hospital, North Western Ethiopia, July 2010

Characteristics	Number (%)
**Age (years)**	
<20	33 (10.8)
20-34	245 (80.3)
35+	27 (8.9)
**Residence**	
Urban	241 (79.0)
Rural	64 (21.0)
**Religion**	
Orthodox	260 (85.2)
Muslim	39 (12.8)
Other	6 (2.0)
**Ethnicity**	
Amhara	296 (97.0)
Other	9 (3.0)
**Marital status**	
Single	13 (4.2)
Married	286 (93.8)
Divorced/Separated	6 (2.0)
**Educational Status**	
Cannot read and write	69 (22.6)
Read and write only	4 (1.3)
Elementary	56 (18.4)
Secondary	88 (28.9)
College level	88 (28.9)
**Occupation**	
Housewives	163 (53.4)
Government employee	62 (20.3)
Merchant	35 (11.5)
Student	18 (5.9)
Daily laborer	13 (4.3)
Jobless	14 (4.6)
**Family size**	
≤3	206 (67.5)
4-6	83 (27.2)
7+	16 (5.3)

Almost half of the mothers (53.4%) were primiparous. Of the multiparous (n=142) women, 107 (81.7%) delivered more than three years ago. A satisfactory number of participants (86.2%) had history of ANC follow up, of which 181 (59.1%) had at least four visits during the current pregnancy. Nearly half (46.2%) of women said they were given dietary counseling during the current pregnancy and 57.7% were taking additional nutrients during pregnancy (Table 2).

**Table 2:** Medical and obstetric profile of study participants at Gondar University Hospital, Northwest Ethiopia, July 2010

Variable	Number (%)
Parity*	
1	163 (53.4)
2-4	120 (39.3)
>4	22 (7.2)
**Birth Space in years**	
<3	24 (18.3)
3-4	39 (29.8)
5+	68 (51.9)
**Small baby at previous birth**	
Yes	15 (10.7)
No	125 (89.3)
**Pregnancy type**	
Wanted and planned	220 (72.1)
Wanted but unplanned	47 (15.4)
Unwanted and unplanned	38 (12.5)
**Number of ANC visits during this pregnancy**	
0	42 (13.8)
< 4 times	82 (26.9)
4+	181 (59.3)
**Trimester of first ANC visit**	
1^st^	44 (16.7)
2^nd^	184 (70.0)
3^rd^	35 (13.3)
**Contraceptive before this pregnancy**	
Yes	208 (68.2)
No	97 (31.8)
**Dietary counseling during pregnancy**	
Yes	141 (46.2)
No	164 (53.8)
**Took additional nutrition during pregnancy**	
Yes	179 (57.7)
No	126 (42.3)

* If a women is her first delivery, she was included as parity=1

Of the 305 deliveries (52.1% males and 47.9% females), about 80% (n=244) were spontaneous vaginal deliveries and 16.4% (n= 50) by caesarian section and the rest 3.6% (n= 11) were assisted deliveries for example by vacuum and forceps. Two hundred fifty one (82.3%) of the mothers knew their LNMP or had it recorded on their follow up chart. For the rest 54(17.7%) the ultrasound estimate was taken from their follow up charts.

However, a total of 23 women failed to remember their LNMP and had no ultrasound gestational age estimates documented, hence they were excluded from further analysis to maintain the unbiased effect of gestational age. Babies of the 23 excluded women had a mean birth weight of 2931 grams and it was not significantly different from the 305 included for further analysis (X^2^=1.78 & p=0.09).

Majority (80.1% or n=260) of newborns from those mothers who remembered their LNMP were delivered at term, 31(12.4%) post term (after 42 completed weeks of gestation) while the rest 19(7.6%) were preterm ones (delivered before 37 completed weeks of gestation). Maternal obstetric and medical illnesses during pregnancy were also assessed for association with LBW. A total of 64 women had either medical or obstetric problems during pregnancy. Of the 44 (14.4%) women with medical problems 13 had HIV/AIDS and 11 had malaria. Similarly out of the 19 (6.2%) women with obstetric related problems 6 women had pre-eclampsia and 2 had eclampsia.

The mean and standard deviation of the birth weights was 2972±476 grams (range 1500-4800 and median 3000 grams). Overall, 52 newborns (17.1% and 95% CI 13.3%, 21.7%) had LBW. The incidence of LBW was 61.9%, 14.3% and 9.4% among the preterm, term and post-term babies respectively.

Female newborns were having smaller birth weight than males (21.5% vs 12.6%) though the difference was statistically significant in the bivariate analysis only (COR=1.95, p-value=0.03). The results of multiple logistic regression analysis (Table 3) indicated a significant association between lack of ANC follow up and LBW. Mothers who had no history of ANC follow up were almost three times (AOR=2.85 & 95%CI 1.10, 7.40) as likely to deliver LBW babies when compared to those who had at least one ANC follow up. Additionally, the number of ANC visits was also one of the factors (AOR=.78 & 95%CI; .61, .99). For each ANC visit, there was a 21% reduction in the risk of delivering a LBW baby.

**Table 3:** Factors associated with LBW, result from logistic regression analyses.

		LBW				
Predictor Variable		Yes	No	Total	Crude OR (95%CI)	Adjusted OR(95% CI)
Sex of the newborn	Male*	20	139	159	1.00	1.00
	Female	32	114	146	1.95(1.06,3.59)**	1.97(.87,4.48)
Maternal age	<20	11	22	33	5.60(1.7,18.0)**	2.10(.53,8.07)
	20-34	206	39	245	2.30(.86,6.16)	1.7(.42,10.11)
	35+ *	25	2	27	1.00	1.00
Residence	Urban*	36	205	241	1.00	1.00
	Rural	16	48	64	1.90(.97,3.70)	1.27(.45,3.63)
ANC Follow up	Yes*	37	226	263	1.00	1.00
	No	15	27	42	3.89(1.65,6.70)**	2.85(1.10,7.40)**
Number of ANC Visits					.79(.63,.99) **	.78(.61,.99) **
Parity	Primipara	43	120	163	5.30(2.48,11.32)**	5.68(2.20,14.66)**
	Multipara*	9	133	142	1.00	1.00
Additional nutrient intake	Yes*	24	152	176	1.00	1.00
	No	28	101	129	1.76(.96,3.20)	1.14(.51,2.55)
GA in weeks	<37*	13	8	21	1.00	1.00
	37-42	36	216	252	.11(.04,.27) **	.14(.04,.47) **
	>42	3	29	32	.06(.02,.28) **	.13(.02,.68) **
Illness during pregnancy	No illness*	41	214	255	1.00	1.00
	Malaria	3	8	11	2.02(.52,7.93)	2.56(.66,9.33)
	HIV/AIDS	6	7	13	4.47(1.49,14.43) **	5.18(2.32,20.43) **
	Others+	2	18	20	.58(.11,1.45)	.61(.10,1.84)

* Reference Category

** Significant at p-value <0.05

+ includes 3 women with tuberculosis, 5 with urinary tract infection, 4 with anaemia and 4 had intestinal parasitosis.

There was a statistically significant difference in the birth weights between primiparous (AOR= 5.68& 95%CI2.20,14.66) and multiparous mothers. Five of the eight (62.5%) mothers with pre-eclampsia/eclampsia delivered LBW babies. However, owing to the small number of the outcome of interest in each disease category, associations were not tested. More than half (53.8%) of the HIV positive mothers delivered LBW babies. HIV positive mothers had a fivefold increased likelihood of delivering LBW babies (AOR=5.18 & 95%CI 2.32,20.43) when compared to HIV negative mothers. In the multivariate analysis factors such as maternal age, residence and sex of the newborn were not significantly associated with LBW (Table 3).

## Discussion

The incidence of LBW in this study (17.1%) is within the range of the country's 2005 DHS values of LBW by measurement (14.6%) and by maternal reports of “very small or small” babies (21.5%) [15]. It is comparable to the average estimate of 16.5% LBW rate for many sub-Saharan countries [2]. But this study was conducted in a tertiary hospital. This result is lower than some other institution based studies in south western Ethiopia (22.5%) and India (24.5%) [16, 17].This difference may be explained by the time gap between these studies and seasons of the year as birth weight may have seasonal variations [18].

However, this study found out a higher prevalence of LBW compared to other hospital based studies in other countries which reported a LBW incidence ranging between 9.0% and 11.8% like Iran, Turkey, Pakistan, Nigeria, Gambia and Ethiopia [8, 10, 12, 19, 20].

In the analysis of associated factors absence or infrequent ANC follow up, first delivery, premature delivery and being HIV positive were independently and significantly associated with LBW. The odds of no ANC follow up among mothers who delivered LBW babies is about 3 times higher than those who had normal weight babies. This may be ascribed to the routine provisions of nutritional and medical advice or care and supplementations offered during ANC visits.

Factors that are associated with LBW such as first time delivery, lack of ANC follow up and being HIV positive were similar to other studies so far reported in Ethiopia [12, 16, 18] and other sub-Saharan African countries[10, 13, 21]. Diseases like malaria, HIV/AIDS and other febrile illnesses cause retarded intra-uterine growth and subsequently lower weight at birth [22]. If the woman does not have ANC follow up illnesses may go undetected and cause more adverse effects on fetal growth.

In the bivariate analysis, maternal age less than 20 years and being female neonate were associated with LBW. However, unlike other studies [18, 19], the association was not significant in the multivariate analysis. This may be due to the interaction effect of parity and age in addition to the low number of LBW cases in the last maternal age category.

The fact that other potential factors considered in this study such as maternal education, marital status, ethnicity, occupation and mode of delivery failed to be associated with LBW can be due to the similar nature of mothers included in the study with majority being illiterate, belonging to one religious or ethnic group.

This study clearly shares the limitations of cross-sectional studies and hence does not show seasonal variations of LBW. Additionally, being a referral hospital the study area can hardly be restricted to Gondar town. This study did not consider some potential risk factors for LBW such as placental factors, congenital syndromes and intra uterine infections.

## Conclusion

In this study, there is a high incidence of LBW, compared to similar hospital based studies in sub-Saharan African. LBW was associated with lack of or infrequent ANC follow up, first delivery and maternal HIV/AIDS. In general, LBW is still a public health problem, so it is essential to provide the necessary facilities for prenatal health care. At the same time, it is necessary to screen pregnant mothers for the important risk factors of LBW such maternal HIV/AIDS and other medical/obstetric problems to provide them with essential prenatal health care services. Efforts to enhance national ANC utilization should be encouraged.

For further research on LBW in this country, it is recommended that other factors be considered, such as home delivery, maternal anthropometry and seasonal variations.
